# Flavonoid-Based Cocrystals: A Comprehensive Study on Their Synthesis, Characterization, Physicochemical Properties and Applications

**DOI:** 10.3390/molecules30214315

**Published:** 2025-11-06

**Authors:** Urszula Izabela Maciołek, Małgorzata Kosińska-Pezda, Tamara Martínez-Senra, Sonia Losada-Barreiro, Carlos Bravo-Díaz

**Affiliations:** 1 Analytical Laboratory, Institute of Chemical Sciences, Faculty of Chemistry, Maria Curie-Sklodowska University, 20-031 Lublin, Poland; 2 Department of Inorganic and Analytical Chemistry, Faculty of Chemistry, Rzeszow University of Technology, 35-959 Rzeszow, Poland; m.kosinska@prz.edu.pl; 3 Departamento de Química-Física, Facultad de Química, Universidade de Vigo, 36310 Vigo, Spain; tamartinez@alumnos.uvigo.es (T.M.-S.); sonia@uvigo.es (S.L.-B.)

**Keywords:** flavonoids, cocrystals, cocrystallization, coformers, non-covalent interactions, bioavailability

## Abstract

Flavonoids are naturally occurring compounds with reported anticancer, antimicrobial, anti-inflammatory, cardio-protective and antioxidant effects. They are increasingly incorporated in functional foods designed to promote health, enhance well-being, and support physical performance. However, their practical use is limited because of their low water solubility and poor absorption within the body. An effective strategy for developing new flavonoid-based formulations involves their transformation into molecular complexes (cocrystals) through cocrystallization, a method that has emerged a powerful tool to modulate the physicochemical and biological properties of polyphenols and other relevant drugs. Cocrystals are stabilized through non-covalent interactions, which can introduce new physicochemical properties to the original molecules (coformers) while retaining the chemical properties of the coformers, as no bonds are broken or formed. Flavonoid-based cocrystals can be obtained through a variety of methods using different coformers, and we aim here to review cocrystals containing flavonoids and coformers, with a focus on their methods of synthesis, physicochemical and biological characteristics, as well as their potential applications in both the food and pharmaceutical sectors.

## 1. Introduction

### 1.1. Flavonoids and Their Interest to Food and Pharmaceutical Sciences

**Figure 1:**
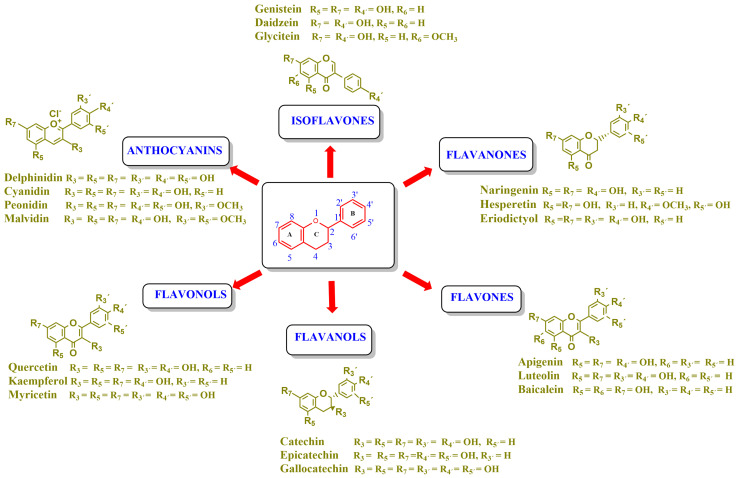
Chemical structures of some representative flavonoids.

Flavonoids are a group of natural and synthetic products, some of which operate as secondary metabolites in higher plants. Natural sources abundant in flavonoids include berries, citrus fruits, grapes, cherries, dock, arugula, onions, artichokes, soybeans, cowpeas, black beans, parsley, oregano, and tea [1]. Most naturally occurring flavonoids possess three hydroxyl groups: two located on the A ring at the C5 and C7, and one at the C4′ on the B ring, Figure 1 [2]. Based on the number of carbon atoms in the C ring, to which the B ring is attached, as well as the degree of saturation and oxidation of the C ring, flavonoids are classified into different subgroups, Figure 1. This extensive group of compounds includes subclasses such as flavones, flavonols, flavanones, flavanols (catechins), anthocyanins, and isoflavones [3,4,5,6].

Flavonoids play key roles in the biological processes of plants, animals, and microorganisms, and interest in their application has grown considerably. Researchers are increasingly exploring their incorporation into novel food products designed to offer both nutritional and health-promoting benefits [6,7]. Table 1 summarizes several of the major pharmacological activities that support the relevance of flavonoids as functional dietary components.

In the plant kingdom, flavonoids contribute to the vivid coloration of flowers and fruits, aiding in the attraction of pollinators and thereby facilitating reproduction through seed and spore germination. Beyond their role in reproduction, they serve as protective agents against a wide array of biotic and abiotic stresses. Their functions include acting as natural UV filters, signaling molecules, allelopathic agents, phytoalexins, detoxifying substances, and antimicrobial defenses [3,4,8]. In humans and animals, flavonoids have been associated with numerous health benefits, including antioxidant, anti-inflammatory, and cardioprotective effects. Ongoing research continues to investigate their potential in disease prevention, therapeutic interventions, and chemoprevention, highlighting their significance in both nutrition science and medical applications [4,8].

**Table 1:** Main biological activities of selected flavonoids.

Flavonoid	Biological or Pharmaceutical Properties	References
cyanidin	antioxidant, antibacterial, anti-inflammatory, antifungal, anti-cardiovascular	[9,10]
catechin	antioxidant, antiviral, anti-inflammatory, anti-cardiovascular	[11,12]
naringin	antiviral, antioxidant, anti-inflammatory, anti-cardiovascular	[13,14,15]
hesperidin	anti-inflammatory, anti-cardiovascular, antiviral	[16,17]
apigenin	antibacterial, antifungal, antiviral	[18,19]
baicalin	anti-cardiovascular, antibacterial, antifungal	[20,21]
luteolin	anti-inflammatory, anti-cardiovascular, antiviral	[22,23]
quercetin	antioxidant, antifungal, anti-inflammatory, anti-cardiovascular, antibacterial	[24,25]
kaempferol	antioxidant, antibacterial, antiviral, anticancer	[26,27]
myricetin	anti-inflammatory, anti-cardiovascular, antioxidant	[28,29]
genistein	anticancer, antioxidant, antifungal, antiviral	[30,31]

The widespread application of flavonoids in food, nutraceutical, and pharmaceutical fields is limited because of their inherent chemical and biophysical characteristics. These include their low aqueous solubility, limited chemical stability, poor bioavailability, and unfavorable pharmacokinetic profiles arising from metabolic transformations in the liver, intestine, and gut microbiota [8,32,33]. Such constraints hinder their effectiveness not only in functional food formulations but also in the development of oral drug delivery systems, where solubility and stability are critical for therapeutic efficacy.

Cocrystallization has emerged as a promising strategy to address these limitations by modifying the physicochemical properties of flavonoids without altering their intrinsic biological activity. By improving parameters such as solubility, dissolution rate, stability, and bioavailability, cocrystallization can significantly enhance the performance of flavonoids in diverse applications. In the food sector, this enables their use as Active Food Ingredients (AFIs) with improved functional properties, while in pharmaceuticals, it opens new opportunities for the formulation of more effective and stable dosage forms, including tablets, capsules, and controlled-release systems. This dual potential positions cocrystallization as a valuable tool in both nutrition science and drug development, bridging the gap between natural product chemistry and practical applications [34].

Cocrystal development is an inherently interdisciplinary field with significant relevance to both the food and pharmaceutical industries, and in this review we aim to explore the potential of flavonoid-based cocrystals by providing a comprehensive overview of the fundamentals of their preparation, characterization and potential applications as delivery systems in pharmaceutical formulations. Topics that are covered include strategies for coformer selection, synthesis methodologies, characterization techniques, and some discussion on advantages, limitations, and challenges. Particular attention is given to the effective enhancement of the physicochemical properties of flavonoids through cocrystallization, as well as their practical applications in a variety of relevant economic sectors.

### 1.2. Cocrystals: Supramolecular Assemblies and Principles of Non-Covalent Interactions

Cocrystallization is a supramolecular phenomenon in which two or more distinct chemical entities assemble into a single crystalline network through non-covalent interactions. In a broader sense, the concept encompasses both the study of multi-component crystalline solids and the principles guiding their rational design. Although there is no strict consensus in the scientific community on what exactly constitutes a cocrystal, cocrystals have become increasingly popular as a potential new or alternative formulations in the solid state for nutraceuticals, pharmaceuticals, and other compounds relevant to various industrial sectors over the past few decades.

The term cocrystal generally refers to a crystalline material composed of two or more distinct molecular species incorporated into the same crystal network. These solids can be described as highly ordered, three-dimensional assemblies in which molecular arrangement is governed by both crystallographic symmetry and specific intermolecular interactions, which in turn dictate their physicochemical properties. Cohesion within cocrystals is mediated by a variety of non-covalent forces, including hydrogen bonding, π–π stacking, van der Waals forces, electrostatic interactions, and halogen bonding. Among these, hydrogen bonding is often the dominant interaction in cocrystal formation, particularly when the components act as weak acids and/or bases. In addition to linking molecular units, hydrogen bonds impart structural directionality and dimensionality to the crystal framework [35,36]. π–π interactions are common in systems containing aromatic rings, arising from attractive forces between electron-rich and electron-deficient regions of aromatic π-systems; these may occur in offset face-to-face or edge-to-face arrangements. Van der Waals interactions, though weaker individually, are omnipresent in molecular crystals, minimizing void space and contributing to overall network stability through close surface contact [37].

In cocrystals, the arrangement of intermolecular interactions reflects both the chemical principles underlying molecular recognition and the spatial constraints imposed by crystal packing. Within this framework, the term “supramolecular synthons” is used to describe recurring structural motifs formed through noncovalent interactions, where certain functional groups selectively associate with complementary ones. This selective association allows researchers to identify suitable coformers capable of modifying the desired physicochemical properties of the cocrystal.

### 1.3. Importance of Cocrystals in Food and Pharmacy

Cocrystals offer the advantage of preserving the structural integrity of the parent molecules until their intended use, thereby reducing degradation processes such as oxidation and photo-oxidation. They can significantly enhance key physicochemical and mechanical properties, including solubility, wettability, flowability, stability, anti-caking behavior, permeability, bioavailability, hygroscopicity, and hardness, and may even mask undesirable tastes [37,38]. The performance of these supramolecular, non-covalent crystalline assemblies is influenced by both the intrinsic nature of their constituent components and extrinsic environmental factors such as temperature, solvent, and pH.

A review of the literature reveals that the majority of cocrystal studies have focused on improving the properties of pharmaceutical compounds, with food-related applications receiving comparatively less attention.

The scope of cocrystallization research area has recently broadened to encompass applications in food science, largely in response to the food industries that are increasingly focusing on the development of functional foods aimed at enhancing the nutritional profile, safety, and overall quality of the food products. Such investigations are particularly relevant in the current context of consumer demands for healthier options. However, the practical integration of relevant bioactive compounds—most notably, the addition of flavonoids—remains challenging, as their physicochemical characteristics considerably constrain their technological applicability and commercial utilization.

Flavonoids open new doors for their use and patentability as they bear in their chemical structures a large number of functional groups that can be efficiently employed to prepare flavonoid-based cocrystals (see Figure 1). The theoretical number of potential coformers is almost unlimited. Nevertheless, a logical, judicious, selection of coformers will help in developing rational protocols for cocrystal preparation and in saving research costs [39,40]. Knowledge of the geometry, number of hydrogen-bond donors, and acceptors of flavonoids, in combination with a knowledge of robust supramolecular synthons, can offer reliable tools for constructing supramolecular, food-relevant architectures [36]. Moreover, molecular recognition between hydroxyl groups and hydrogen-bond donating groups (carboxyl, carbonyl, amide, etc.) has been reported as the main synthon behind the formation of cocrystals.

### 1.4. Coformer Screening in Cocrystal Design

Appropriate coformers are crucial in the successful formation of flavonoid- and polyphenol-based cocrystals, as they strongly affect the crystal structure and, in turn, can modulate key physical properties such as solubility, stability, and bioavailability [41,42]. Furthermore, coformers contribute to structural stabilization through non-covalent interactions including hydrogen bonding, π–π stacking, and van der Waals forces, ultimately improving the performance of active compounds compared to their raw forms [41,42].

The selection of an appropriate coformer can be approached through two broad approaches: (1) experimental techniques and (2) a range of knowledge-based methods. The latter includes hydrogen-bonding propensity rules, pK_a_-based prediction models, Cambridge Structural Database [43,44] (CSD) screening, supramolecular synthon compatibility assessments, lattice energy calculations, Hansen solubility parameters, thermal analysis, saturation temperature measurements, and virtual cocrystal screening [45,46,47]. Each method offers unique insights, though computational strategies such as mixing enthalpy calculations or molecular electrostatic potential comparisons may yield divergent results depending on the theoretical model, and should therefore be complemented with experimental validation [36,48].

The presence of hydroxyl groups in flavonoids, Figure 1, facilitates hydrogen bonding, making them suitable candidates for forming supramolecular synthons—structural motifs within cocrystals that arise from predictable intermolecular interactions. These synthons can be classified as homosynthons (involving identical self-complementary functionalities) or heterosynthons (formed by different but complementary groups), with the latter often being more robust. Analyzing the frequency of specific hydrogen bonds between functional groups in the CSD can help identify coformers with high likelihood of interaction, aiding rational design [39].

Molecular interactions between hydroxyl groups and hydrogen-bond donating groups such as carboxyl, carbonyl, amide, and pyridyl have been reported as the main synthon for the formation of polyphenol-based cocrystals [39,40]. These intermolecular interactions should be determinant in the search for potential flavonoid-based coformers, hence the search for optimal coformers can be carried out by analysing the main intermolecular interactions of the structures reported in the literature, paying special attention to those of well-characterized, solid-state, flavonoid-based cocrystals.

Flavonoid and coformer molecules can be connected by a variety of hydrogen bonds such as N…H–O, N–H…O, O…H–O, O–H…O [49,50,51], S…H–O [52], C–H…O [50,52,53,54] and O–H…Cl^−^ [55,56]. In addition to hydrogen bonds, long-range van der Waals forces and π-stacking interactions may be also important as these dispersive interactions play a key role in the stacking and packing of layers in the 3D architecture of the crystals. The stacked layers create the three-dimensional framework of the flavonoid cocrystal structure stabilized by π-stacking contacts (including face-to-face, offset face-to-face and edge-to-face π…π stacking) [57,58], π-stacking, and van der Waals forces [59], C–H…π-stacking [50,60].

The safety of selected coformers is essential in food and pharmaceutical applications. Only substances recognized as non-toxic should be used, with reference to authoritative databases such as the U.S. FDA’s “Substances Added to Food” (formerly EAFUS), the “Generally Recognized as Safe” (GRAS) database, and the European Regulation (EC) No. 1333/2008 on food additives [39]. Coformer selection should also be guided by the intended application and desired modifications in the cocrystal’s behavior. For instance, pyridine has been shown to enhance thermal stability and solubility [61], 4,4′-azobispyridine can enable photoresponsive behavior through its azo group [62], while compounds like caffeine or betaine may function as adjuvants or flavoring agents. In pharmaceutical applications, coformers such as temozolomide have potential for targeted activity against diseases like glioblastoma multiforme [63].

In conclusion, the design of flavonoid-based cocrystals relies heavily on both the functional compatibility and safety profile of the coformer. A balanced combination of computational prediction, experimental validation, and regulatory awareness ensures the development of effective and safe cocrystals tailored for specific functional outcomes.

### 1.5. Key Supramolecular Synthons in Polyphenol-Based Cocrystal Formation

Proper identification of suitable coformers can be guided by examining the primary intermolecular interactions observed in previously reported flavonoid-based cocrystals, focusing exclusively on well-characterized solid-state structures documented in the literature. This methodology was chosen according to the following criteria:

(1) Structural precision, since flavonoid-based cocrystals reported in the literature with strictly defined 3-dimensional configurations provide highly accurate data regarding the spatial arrangement of flavonoids and coformers, as well as on the nature of inter- and intramolecular interactions. Such criteria should facilitate the unambiguous identification of key intermolecular interactions defining the cocrystal stability and its physicochemical properties.

(2) Property predictability, since the use of cocrystals with well-defined structural frameworks allows for the systematic investigation of how specific molecular interactions influence macroscopic properties, including solubility and thermal behavior. This predictability is of paramount importance for the rational design of cocrystals tailored to exhibit desired physicochemical characteristics.

(3) Utility in material design—a comprehensive understanding of intermolecular interactions in structurally well-characterized cocrystals serves as a foundation for the rational development of novel materials. This is particularly relevant in those cases where the optimization of flavonoid stability, solubility, and bioavailability can be achieved through strategic engineered cocrystallization.

In searching the Cambridge Structural Database (CSD) database we found that, in the past 7 years, the number of reports (>100) providing well-defined solid state structures is representative enough to permit a thorough analysis and to extract some important conclusions [43]. For convenience, according to the division proposed by E. Grothe [64], the flavonoid cocrystals were classified into: cocrystals, solvated cocrystals (cocrystals containing two or more coformers and one or more solvent molecules), cocrystal salts (cocrystals containing at least two ions, no solvent molecules), salts (crystal containing at least two ions), and salt solvates (cocrystals containing no coformers but one or more solvent molecules and two or more ions). In the analyzed period, we found that 33 solvated cocrystals, seven cocrystals salts, two salts, and three salt solvates with well-defined crystal structures were reported [43].

Intermolecular interactions were analyzed using the Mercury software (version 2025.2.0), which systematically retrieves structural information from well-defined entries in the Cambridge Structural Database (CSD). The program allows for the identification and quantification of specific interactions between functional groups, providing insight into their type, frequency, and spatial orientation. Application of this approach to flavonoid–coformer/solvent systems revealed that the -OH and carbonyl groups display an uneven distribution of interactions with coformer molecules, highlighting the preferential sites for molecular recognition and the factors influencing cocrystal assembly.

**Figure 2:**
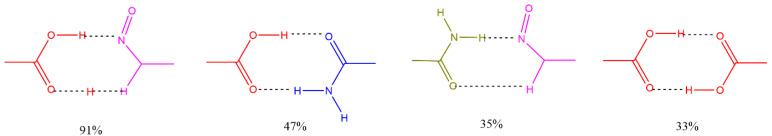
Common supramolecular synthons formed from carboxylic acids, amides, pyridines, and other aromatic nitrogens and their frequency of occurrence in the structures reported in CSD.

Figure 2 shows some of the main interactions occurring in cocrystals and their frequency of occurrence in the CSD. The main functional groups involved are 7OH, 3′OH and 4′OH, located at the periphery of flavonoid rings A and B. Table 2 shows, for selected flavonoids, the main functional groups and number of well-defined structures found in the CSD in the form of cocrystal solvates, cocrystal salts, salts, and salt solvates.

These intermolecular interactions should be determinative in the search for potential flavonoid-based coformers. A thorough analysis of the interactions indicates that coformer molecules interact via functional groups capable of acting as either hydrogen bond donors or acceptors. These molecules commonly contain groups such as amines, amides, carbonyls, carboxyls, pyridyl rings, and other nitrogen- or sulfur-containing heterocycles. Table 3 lists selected coformers identified from the CSD database, along with a brief description of the role of their functional groups in the cocrystal structures, particularly regarding intermolecular interactions. In this context, (aromatic) hydroxyl groups and hydrogen-bond donating moieties—such as carboxyl, carbonyl, amide, and pyridyl groups—have been identified as the primary synthons driving the formation of polyphenol-based cocrystals [39,40].

**Table 2:** Some flavonoid-based cocrystals with well-defined structures that have been reported in the CSD. The number of cocrystal structures, cocrystal solvates, cocrystal salts, salts, and salt solvates found for a given flavonoid are also indicated. The functional groups of the flavonoids that most frequently participate in intermolecular interactions with the functional groups present in coformer molecules are highlighted in bold green color.

SUBCLASS	Flavonoids	Functional Groups of Flavonoids Involved in Hydrogen Bonding with the Coformer/Solvent	Cocrystals	Cocrystal Solvates	Cocrystal Salts	Salts	Salt Solvates	References
**FLAVONE**	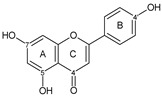 apigenin	5OH **7OH** 4C=O **4′OH**	6	2	-	-	1	[39,65,66,67]
**FLAVONOL**	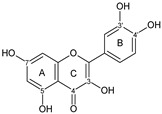 quercetin	3OH 5OH **7OH** 4C=O **3′OH** **4′OH**	11	7	-	-	-	[60,68,69,70,71,72,73,74,75,76]
**FLAVANONE**	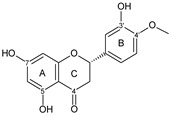 hesperetin	5OH **7OH** **3′OH**	10	-	4	1	-	[55,57,59,60,77,78,79,80,81,82,83]
**FLAVANONOL**	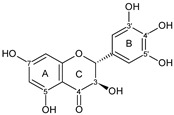 dihydromyricetin	3OH **7OH** **3′OH** **4′OH** **5′OH**	4	6	1	-	-	[51,56,60,84,85,86,87]
**ISOFLAVONE**	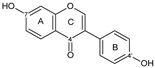 daidzein	**7OH** **4′OH**	2	-	-	-	1/0	[54,84,88]
	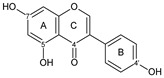 genistein	**7OH** 4C=O **4′OH**	6	-	-	-	1/0	[60,77,80,89,90,91]
**CHALCONE**	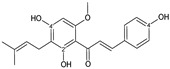 xanthohumol	**4OH**	3	1	-	-	-	[92,93]
**CATECHIN**	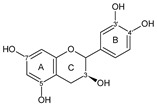 catechin	3OH **7OH** **3′OH** **4′OH**	1	1	-	-	-	[94]

**Table 3:** Selected coformers involved in the formation of cocrystals with flavonoids. The functional groups of the coformers that act as hydrogen bond acceptors are highlighted in green, those acting as hydrogen bond donors in red, and the functional groups that are both acceptors and donors in blue colors. The digits in parenthesis indicate the number of structures found in CSD containing the particular coformer (accessed in January 2025).

Coformers with functional groups acting as hydrogen bond acceptors with flavonoids	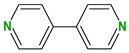	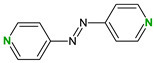	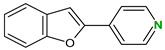	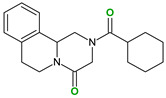
	4,4′-Bipyridine (14)	4,4′-Azobispyridine (5)	4-(1-Benzofuran-2-yl)pyridine (1)	Praziquantel (3)
	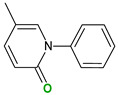	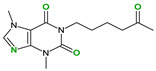	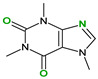	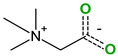
	Pirfenidone (2)	Pentoxifylline (2)	Caffeine (7)	Betaine (4)
Coformers with functional groups acting as hydrogen bond acceptors and/or donors with flavonoids	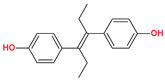	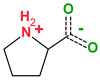	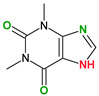	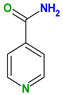
	Diethylstilbestrol (4)	Proline (3)	Theophylline (7)	Isonicotinamide (5)
	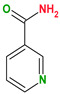	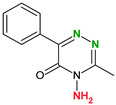	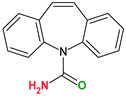	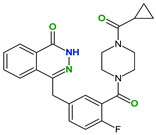
	Nicotinamide (4)	Metamitron (2)	Carbamazepine (6)	Olaparib (2)
Coformers capable of forming cocrystals, salts cocrystals and salts with flavonoids	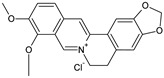	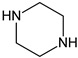	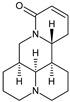	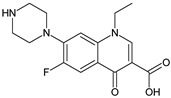
	Berberine chloride (5)	Piperazine (4)	Sophocarpine (1)	Norfloxacin (1)

## 2. General Theoretical Background on the Preparation of Flavonoid-Based Cocrystals

Before depicting the intricate rationale for the general development of cocrystals, it is crucial to analyze the defining characteristics that make them important and unique, mainly because currently there is no technology or methodology capable of predicting whether the preparation of cocrystals will be successful or not. In many aspects, materialization of cocrystals remains more of an art than of a science, and significant research efforts are made in multidisciplinary areas [39,45,95,96].

Formation of cocrystals cannot be (formally) considered as a chemical reaction since no bonds are broken or formed but only intermolecular forces are involved in their formation. However, some of the models developed to interpret chemical reactivity, and in particular the transition state theory [97,98], can be useful as a starting point to gain insights into the theoretical background needed for a successful preparation.

**Figure 3:**
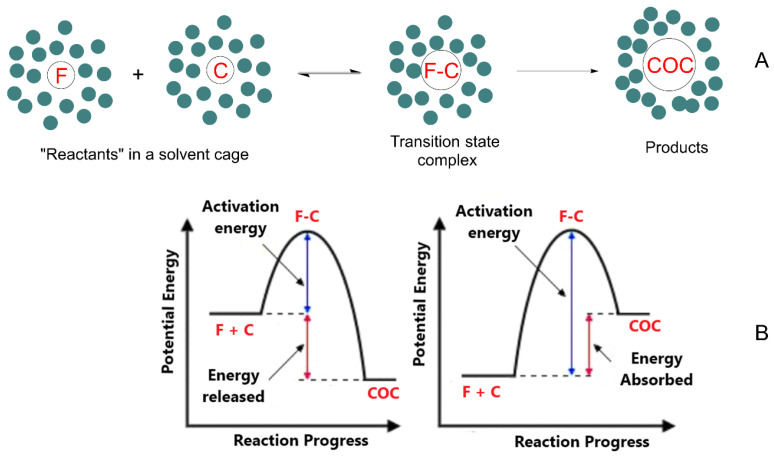
(**A**) Flavonoid-based cocrystal formation requires that the solvated flavonoid F and coformer C diffuse through the solvent to form an “encounter pair” F–C in a solvent cage. If the energy of the reactants is high enough to overcome the activation energy, *E*_a_, the cocrystal COC will form, otherwise F–C will separate and cocrystal formation will not occur. In panel (**B**), the figure on the left is representative of an exergonic process, while the one on the right represents an endergonic process.

The parent compounds (flavonoid and coformer) are initially dissolved in a given solvent where they are solvated, e.g., the molecules are surrounded by a solvent cage. According to transition state theory, the formation of the cocrystal requires that both components approach each other forming an “encounter pair” surrounded by the solvent molecules. If the energy of the reactants is high enough, the cocrystal can be formed, otherwise, the reactants separate and do not react. This process is illustrated in Figure 3.

Thus, two factors govern cocrystal formation: thermodynamics (energy) and kinetics (time). From an energetic point of view, the formed cocrystals (COC) can be more stable than the parent compounds (exergonic process) or less stable (endergonic process). Their formation, however, requires a minimum energy that the reactants should have (equal to the activation energy *E*_a_) for their cocrystallization. Therefore, even if cocrystal formation is energetically favorable (e.g., an exergonic process), the activation energy of the process should be low enough to allow cocrystallization. In contrast, if the activation barrier is too high, cocrystal formation will not take place in practice. These scenarios can be illustrated with common conceptual energy–reaction coordinate diagrams, showing that when the reactants approach each other, the potential energy increases up to a maximum (transition state, Figure 3) [97]. A key point necessary to understand cocrystal formation lies in understanding the thermodynamics of cocrystals, i.e., to gain knowledge on the difference in energy between the reactants and the cocrystal. As predictions are generally arduous, network energies are usually employed for cocrystallization prediction by assuming that the variation in enthalpy is the major contributor to the Gibbs free energy [47,99,100].

Molecules in the transition state rearrange to form a thermodynamically more stable cocrystal provided that the energy of the reactants is enough to overcome the energy barrier (usually calculated according to the Arrhenius equation). Thus, reaction conditions (temperature, viscosity, pressure, etc.) must be adequate. It becomes clear that if reactants do not have enough energy to overcome the energy barrier, cocrystallization will never happen as the energy barrier will be high enough to minimize nucleation.

It follows from the above discussion that some external energy input is necessary to overcome the energetic barriers associated with the formation of cocrystals, particularly if flavonoids and coformers exhibit low binding affinity, or if they have very different solubilities that make difficult to reach the adequate stoichiometry. Increasing the kinetic energy of the reactants may favor cocrystallization, and it has been suggested that rapid reaction conditions are required for the successful preparation of elusive cocrystals [48]. In some instances, high energy barriers can be circumvented by using catalysts. For example, upon incorporation of heteronuclear seeds [48,101], the structural resemblance may be tailored, reducing the energy required for cocrystallization. Production of cocrystals can also be facilitated by introducing a third component such as fatty acids or polymers that alters the reaction conditions. Although no single method exists to predict the successful preparation of cocrystals, different prediction tools such as hydrogen-bond propensity, molecular complementarity, and a thorough analysis of the most common coformers may help in current cocrystal preparation methodologies [39,45,48,96].

## 3. Laboratory Strategies for Preparing Cocrystals and Techniques Employed for Their Characterization

Any optimal approach to the synthesis of flavonoid-based cocrystals (and, more broadly, other polyphenols) aims to (1) obtain both cocrystal powders and single crystals, which enables precise structural characterization, and (2) study their physicochemical properties. But both of these goals are not always achieved at the same time or by employing the same methodologies [39,51,68,77,102]. The method of obtaining powders is mainly aimed at searching for the most optimal and fast path to obtaining cocrystals, which can be used for further research and analysis, while the synthesis of a single crystal is exclusively aimed at confirming the exact cocrystal structure obtained in the powder form.

**Figure 4:**
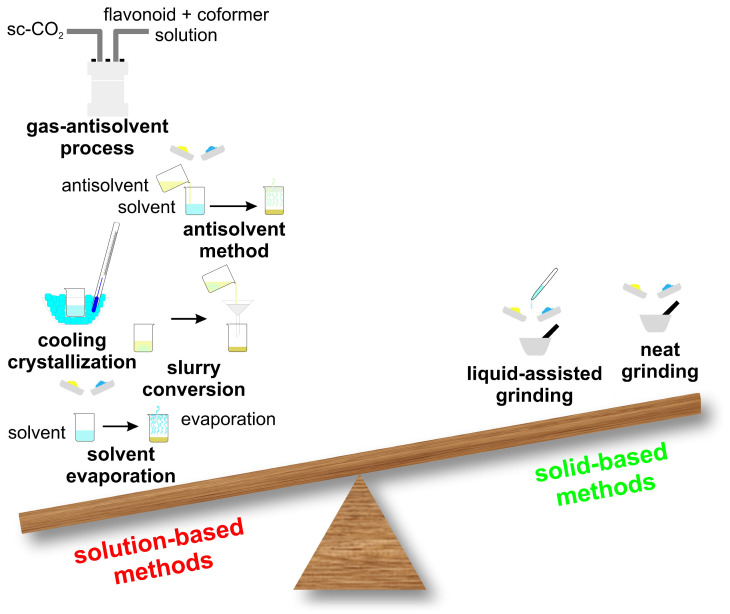
Some representative solution-based and solid-based methods for cocrystal formation.

**Figure 5:**
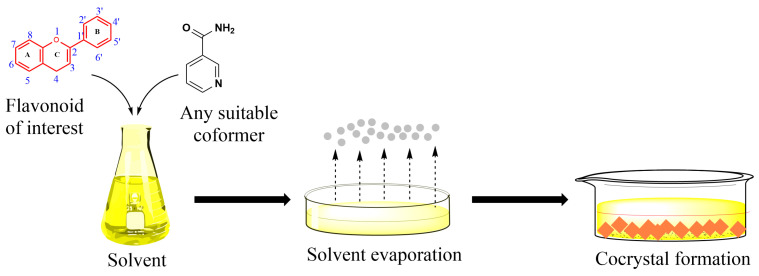
Simplified sequence illustrating a solvent-based method employed in the preparation of cocrystals.

Choosing an appropriate cocrystallization method is still largely empirical and, in many cases, depends on the researcher’s experience and iterative trial-and-error experiments. Two major experimental approaches are found in the literature to prepare cocrystals: solid-state and solvent-based methods (Figure 4). In the scientific literature, the majority of reported pharmaceutical cocrystals have been obtained using solution-based synthesis methods, as they typically allow for better control over crystallization and phase purity. However, solid-state techniques, such as neat grinding or liquid-assisted grinding, are increasingly recognized as more environmentally friendly approaches, since they minimize or even eliminate the need for organic solvents. In addition to their ecological advantages, solid-state methods are often faster, simpler to perform, and more easily scalable, making them an attractive alternative for sustainable cocrystal production. An example of a solution-based cocrystal synthesis method is illustrated in Figure 5 [52,53,56,65,66,68,74,75,80,84,85,102,103,104,105,106,107,108,109].

Solid-state techniques, such as dry grinding, couple mechanical and chemical processes at the molecular level. These methods are highly effective for cocrystal preparation, as the mechanical energy applied induces fractures and stress within the starting materials. This not only increases their surface area but also enhances the number of surface molecules available for interpenetration, thereby promoting molecular contact and facilitating cocrystal formation. The most frequently employed solid-state methods are neat grinding and liquid-assisted grinding. In recent years, hot-melt extrusion cocrystallization techniques have been proposed as new methods for cocrystallization [110,111,112].

Solvent-based methods, Figure 5, have been widely used for synthesizing cocrystals and constitute the majority of the methods currently employed. Solvents play a crucial role affecting various cocrystal properties including their shape, solvate formation tendency, polymorphism and purity. Solvents are usually selected on the basis of the differential solubility of the starting materials (e.g., flavonoid and coformer): ideally, the solubility should be similar, because if they differ too much, the method is not effective [113,114].

Neat-grinding was the method of choice to prepare cocrystals of flavone with naringenin [49], baicalein with metamitron [104], quercetin with benzamide, picolinamide, isonicotinamide and pyrazinoic acid [115], naringenin with metamitron [104], 4-(1H)pyridine, piperazine, anthranilamide and 4,4′-bipyridine [49] and catechin with urea [109].

Liquid-assisted grinding is based on the addition of small amounts of solvent (water, methanol, ethanol, isopropanol, acetone, chloroform, etc.) that act as a catalyst increasing the rate of cocrystallization. These solvent drop assisted methods may be as effective as the solid grinding methods, but have additional problems related to disposal and environmental risks. Liquid-assisted grinding was used in synthesis, among others, of cocrystals of chrysin with cytosine, apigenin with *δ*-valerolactam [65] and with nicotinamide [103], quercetin with o-dianisidine, 1,10-phenanthroline, 4-(5-ethyl-1-benzofuran-2-yl)pyridine, 4-(1-benzofuran-2-yl)pyridine [74], benzamide, picolinamide, isonicotinamide and pyrazinoic acid [115], levofloxacin and ciprofloxacin [116], pyrazinamide [105], oxaliplatin [106] and levofloxacin [116], oxaliplatin [106], lobaplatin [108], isonicotinamide, theobromine and cytosine [117], and genistein with ligustrazine [53].

Solvent evaporation, together with slurry approaches, are the most widely employed methods in the synthesis of flavonoids cocrystals (Table S1 Supplementary Material). Evaporative cocrystallization is grounded on the supersaturation of the system caused by solvent evaporation. Solvent evaporation is a rather common method in cocrystal formation. Typically, an excess of solvent is employed for solubilizing the stoichiometric ratio of flavonoid (or polyphenol) and coformer. The solvent is then evaporated either at room temperature or by applying a vacuum to accelerate the process of drying. A wide spectrum of used solvents can be used, including acetone, acetonitrile, ethanol, ethyl acetate, methanol, tetrahydrofuran [60,65,66,92,118,119,120,121,122], as well as binary ethanol/ethyl acetate, ethanol/acetone, ethanol/dichloromethane, dichloromethane/isopropanol, dichloromethane/ethyl acetate, water/ethanol, and ternary methanol/ethyl acetate mixtures [60,65,70,86,123,124,125,126].

The slurry method involves cocrystallization from a suspension of the target molecules and coformers. Cocrystals are naturally metastable compared with their parent components, and their formation is driven by high solute concentration favoring nucleation and cocrystal growth. Auxiliary techniques such as ultrasound and microwave may improve the slurry cocrystal conversion. Ultrasound-assisted solution crystallization uses ultrasound pulses to generate voids or air bubbles in liquid while imparting cycles of compression and thinning to the inner part of the liquid. During the compression cycle, air bubble contents are compressed and result in the formulation of cocrystals nuclei during the flavonoid crystallization process. The microwave cocrystallization method exploits microwaves as a heating source, increasing the rate of cocrystal formation. Parent components are placed in a suitable microwave reactor at the desired temperature and pressure for a specific time to obtain the desired cocrystal product, providing a clean, scalable, economical and fast preparation method [127]. Slurry methods were used to prepare a large number of flavonoid-based cocrystals (Table S1 Supplementary Material).

Supercritical fluid technology has been satisfactorily implemented in food cocrystal preparation. The higher the solubility of polyphenols, the higher are the chances of producing cocrystals [128,129]. Supercritical fluid technology has been evolving over the past few years as a consequence of the rapid expansion of supercritical solutions, the use of supercritical anti-solvent crystallization methods, supercritical fluid enhanced atomization, atomization, anti-solvent crystallization, and gas anti-solvent crystallization technologies [128,129]. Sometimes, when pure CO_2_ does not produce cocrystals, small amounts of other solvents such as acetone [130] or ethanol [131] are added to promote cocrystallization.

In the gas antisolvent techniques, pressurized CO_2_ is introduced in a high-pressure chamber containing the solute solution, decreasing the solvent’s ability to solubilize solutes and leading to solute cocrystallization [128]. This method offers some advantages over traditional methods allowing a fast single-step process, providing a suitable system to control particle size and morphology by fine-tuning pressure and temperature. A gas antisolvent process was used to obtain cocrystals of quercetin with nicotinamide [130] and L-proline [131]. Supercritical acetone [130] and ethanol [131] were used as alternative solvents in the preparation of some cocrystals.

A variety of methods are employed to fully characterize cocrystals, which are necessarily connected with investigating their physicochemical properties, e.g., specific surface area determination, solubility (selectivity, saturation studies), preparation of ternary phase diagram, stability studies, food, and biological and health activities (toxicity, antioxidant, antibacterial, antihaemolytic hepatotoxicity, cytotoxicity test, antitumor, etc.) [132,133,134,135]. A literature survey indicates that those methods can be classified in three main groups: (1) power X-ray diffraction, (2) thermal analysis (differential scanning calorimetry), and (3) spectroscopic methods (FTIR, Raman, solid-state NMR, etc.). In any case, it should be emphasized that empirical methods of flavonoids cocrystals investigation are very frequently supported by computational studies.

Both powder diffraction and X-ray methods provide invaluable information about cocrystals. In particular, single crystal X-ray diffraction has become a cornerstone of most cocrystal research programs, validating in some cases crystal engineering strategies based on preferred hydrogen bonding motifs, while highlighting in others the unpredictable nature of molecular self-assembly in complex systems [78,116,120,136]. X-ray diffraction is one of the most popular and precise methods for the identification and quantification of cocrystals. Most frequently, characteristic peaks of the cocrystal are compared with those of parent components (identification purposes), and such changes may also be used for quantitative analysis to estimate the yield of cocrystals and the percentage of parent components in the obtained product.

Thermal analysis methods [58,120,136,137] are very rapid and efficient for the characterization of cocrystals as they require very small amounts of sample to conduct the characterization study. Their main limitation is related to the thermal stability of the materials to be evaluated. They include a series of techniques in which a physical or chemical change is measured as a function of temperature according to a predefined heating or cooling program. Particularly, when studying cocrystals, parameters such as melting points, crystallization, sublimation, and decomposition may be quantified.

Spectroscopic methods [136] exploit electromagnetic radiations of different wavelengths to get insights into the composition and structure of cocrystals. Their principal advantage comes from being non-destructive methods requiring very small amounts of sample for data collection. Spectroscopic methods can be divided into those requiring high magnetic fields (e.g., Nuclear Magnetic Resonance, NMR) and those based on the vibrational properties of the cocrystal components (Infrared, Raman spectroscopies). Solid-state NMR provides very detailed structural information about cocrystals (when compared to spectroscopic or X-ray diffraction methods) proving molecular associations and allowing, in some instances, observation of important structural features such as hydrogen-bonding.

Table S2 (see Supplementary Information) outlines the principal methods used in cocrystal characterization. Comprehensive analysis of flavonoid cocrystals requires evaluation of their physicochemical attributes (e.g., surface area, solubility, phase behavior, stability) alongside assessments of biological and functional properties (e.g., toxicity, antioxidant and antibacterial activity, cytotoxicity, antitumor potential). The most important techniques for cocrystal characterization include diffraction, thermal analysis, spectroscopic methods, as well as complementary approaches such as microscopy and biological assays.

## 4. Applications of Flavonoid Cocrystals

**Figure 6:**
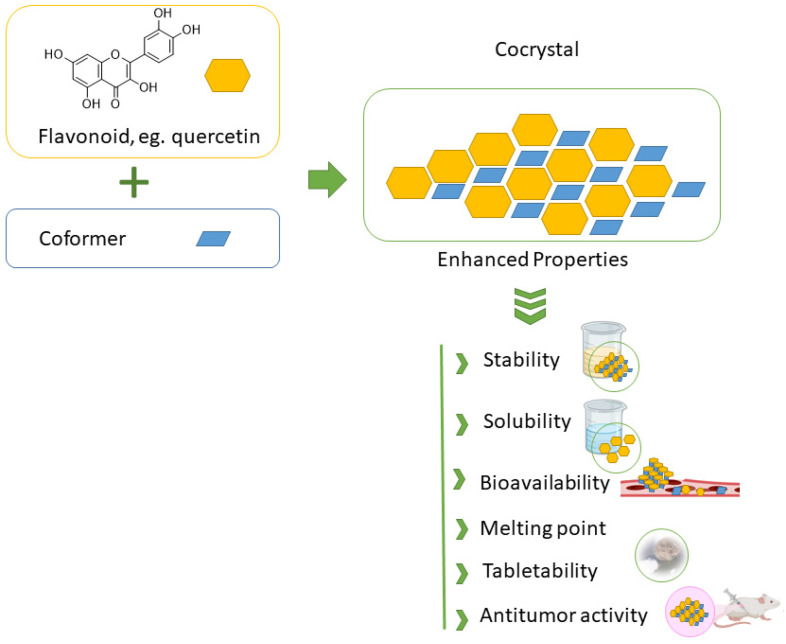
Schematic illustration of cocrystal formation from flavonoids and coformers, resulting in enhanced stability, solubility, and bioactivity.

Many naturally occurring flavonoids suffer from poor aqueous solubility and consequently limited bioavailability, which significantly hampers their clinical and pharmaceutical utility. From the discussion in previous sections, one can easily envisage that flavonoid-based cocrystals exhibit a range of promising properties, particularly in terms of their physicochemical and biological profiles, which are essential for a variety of food and pharmaceutical applications. One of the key motivations behind the development of flavonoid cocrystals is to address these limitations—namely, to enhance solubility and dissolution rates, thereby improving oral bioavailability, Figure 6.

Tokunaga et al. [58] successfully synthesized cocrystals of nobiletin with several coformers, including oxalic acid, gallic acid, salicylic acid, urea, and formamide, using mechanochemical methods. These cocrystals exhibited significantly improved aqueous solubility compared to pure nobiletin, suggesting potential for enhanced therapeutic efficacy, particularly in the contexts of anti-inflammatory, neuroprotective, and anticancer therapies.

Similarly, multiple flavonoid-based cocrystals have shown marked improvements in intrinsic dissolution rate (IDR) or solubility relative to their parent compounds. Some notable examples include: naringenin with isonicotinamide, picolinic acid, and betaine [138]; baicalein with 4,4′-bipyridine [124]; phloretin with isoniazid [119]; betaine with baicalein, phloretin, and quercetin [72]; phloretin with nicotinamide and isonicotinamide [139]; daidzein with piperazine [84]; dihydromyricetin with 4,4′-bipyridine [86]; fisetin with isonicotinamide, nicotinamide, and caffeine [140]; hesperetin with carbamazepine [83] and with piperine [82]; and luteolin with isoniazid and caffeine [141]. All the above studies illustrate how cocrystallization can be strategically employed to optimize the oral delivery of poorly soluble natural products.

Beyond solubility enhancement, cocrystal formation has also been proven effective in modulating the bioavailability and permeability of flavonoids. This is evidenced by the improved properties observed in systems such as chrysin with berberine [142], daidzein with piperazine [84], genistein with tetramethylpyrazine [53], and hesperetin with piperine [82].

Cocrystallization also holds promise in improving the physicochemical stability of flavonoids. For example, cocrystals of dihydroquercetin and dihydromyricetin with 4,4′-bipyridine have shown significantly reduced hygroscopicity [86]. Additionally, the genistein–tetramethylpyrazine cocrystal demonstrates enhanced chemical stability of tetramethylpyrazine, which may contribute to improved shelf life and formulation performance [53].

While the majority of studies focus on solubility enhancement, cocrystallization can also be utilized to reduce solubility in order to achieve sustained-release profiles. For instance, Meng et al. [80] developed cocrystals of pirfenidone with hesperetin and genistein, yielding a slower dissolution profile advantageous for long-acting oral dosage forms. Similarly, cocrystallization of tegafur and myricetin significantly improved tabletability and stability, reduced hygroscopicity, and enhanced compatibility, while offering modified drug release suitable for anticancer therapies [143].

In addition to physicochemical modulation, recent studies have begun to explore the potential of flavonoid cocrystals to influence biological activity. By altering the crystal packing environment, cocrystallization may impact interactions with biological targets, chemical stability, and photophysical properties such as fluorescence and photoluminescence. Notably, the cocrystal of dihydromyricetin with pentoxifylline exhibited a significantly higher inhibitory rate against HepG2 cells than either individual component, suggesting a synergistic anticancer effect [87]. Enhanced antitumor activities were also reported for cocrystals of myricetin, genistein, hesperetin, and pratensol with 4,4′-ethylenebispyridine against HCT-8 and Caco-2 cell lines [77]. Gastrointestinal digestion studies of the phloretin–isoniazid cocrystal revealed improved release and superior antioxidant and cytotoxic activities compared to pure phloretin [119].

Finally, it may be worth noting that pharmaceutical cocrystals act as a bridge between drug discovery and pharmaceutical product development, resulting in a material that is patentable. This is a consequence of their manifold applications. For example, flavonoid-based cocrystal technology has shown promise in improving drug safety profiles. For example, cocrystallization of pyrazinamide (a first-line antituberculosis drug) with baicalein—a flavonoid known for its hepatoprotective effects—resulted in enhanced water solubility, bioavailability, and, notably, a substantial reduction in pyrazinamide-induced hepatotoxicity [105].

## 5. Conclusions and Future Outlook

Cocrystals gained the interest of researchers mainly because of their potential to alter the physicochemical, mechanical, and pharmacokinetic properties of active ingredients without modifying their therapeutic activity. Some of these aspects were discussed in this review, aiming at the evaluation and potential of cocrystallization as an important tool to modulate the properties of polyphenols, and particularly flavonoids, in the context of their applications in the food and pharmaceutical sectors. This increases interest in cocrystal investigation for food product development and promotes the use of natural compounds with anti-cancer, anti-inflammatory, and antioxidant properties that are increasingly used in functional foods designed to improve human health and wellbeing.

The tunable aspects of flavonoid cocrystallization were also addressed, and, after a thorough literature search in the CDS database, we set the basis for coformer screening, widening their application for food and pharmaceutical products. Nevertheless, additional studies are required, as flavonoids, being polymorphic supramolecular synthons, do not exhibit fully predictable behavior. This is due to the presence of a C=O group and up to six –OH groups, which allow for variable intermolecular hydrogen-bond directionality, resulting in considerable flexibility of contacts within crystalline supramolecular frameworks. In multicomponent flavonoid systems, potential coformers may include molecules with functional groups capable of acting as hydrogen-bond donors or acceptors, such as carboxyl, carbonyl, hydroxyl, amine, imide, or heterocyclic moieties containing nitrogen or oxygen atoms. The coformer molecules are bound to flavonoids via hydrogen bonds, most often to the terminal hydroxyl groups of the flavonoid molecule.

The potential of flavonoid-based cocrystals was summarized on the grounds of the use of food- or pharmaceutical-grade coformers, showing that cocrystallization provides an excellent tool to modulate the desirable properties of flavonoids (and, in general, those of other polyphenols), broadening their application in food products as nutraceuticals. We also reviewed some of the most important cocrystallization methods and included some discussion on potential pharmaceutical/medical properties of flavonoids and their potential use as active pharmaceutical ingredients with the aim of expanding the possible applications of these new materials. Cocrystals may provide a solution for the problem of potentially good active pharmaceutical ingredients that suffer from poor bio-availability. The poor predictability of successful cocrystal preparation hampers progress in the pharmaceutical, but also in the cosmetic and food industries (for example, in the preparation of food supplements) as only a few commercial products are available on the market. It appears therefore that much work is needed to improve predictability. The development of molecular dynamics models should produce synergistic effects when employed in combination with techniques commonly employed by crystal engineers, material scientists, and chemists.

## Data Availability

Data are available upon request to any masthead.

## Supplementary Materials

The following supporting information can be downloaded at: https://www.mdpi.com/article/10.3390/molecules30214315/s1, Table S1: Synthetic routes of flavonoid-based cocrystals reported from 2017 to date in the CSD accessed in January 2025) and solvents employed. Table S2: Techniques and methods commonly used to characterize flavonoid cocrystals and physicochemical and biological properties of the cocrystals reported in CSD from 2017 to date.
