# Supplementary material for: Flavonoid-Based Cocrystals: A Comprehensive Study on Their Synthesis, Characterization, Physicochemical Properties and Applications
Source: Molecules. 2025 Nov 6;30(21):4315. doi: 10.3390/molecules30214315 (PMC12608692; doi:10.3390/molecules30214315)
Supplement: Supplementary file 1 [file molecules-30-04315-s001.zip › molecules-3874041-supplementary.pdf]

Supplementary materials

to

FLAVONOID-BASED COCRYSTALS: A COMPREHENSIVE STUDY ON THEIR SYNTHESIS, CHARACTERIZATION, PHYSICOCHEMICAL PROPERTIES AND APPLICATIONS

Urszula Izabela Maciołek [1*], Małgorzata Kosińska-Pezda [2], Tamara Martínez-Senra [3], Sonia Losada-Barreiro [3] and Carlos Bravo-Díaz [3*]

[1]Analytical Laboratory, Institute of Chemical Sciences, Faculty of Chemistry, Maria Curie-Sklodowska University, 20-031 Lublin, Poland

[2]Department of Inorganic and Analytical Chemistry, Faculty of Chemistry, Rzeszow University of Technology, 35-959 Rzeszow, Poland; m.kosinska@prz.edu.pl

[3]Departamento de Química-Física, Facultad de Química, Universidade de Vigo, 36310 Vigo, Spain; tamartinez@alumnos.uvigo.es (T.M.-S.); sonia@uvigo.es (S.L.-B.)

[*]Correspondence: urszula.maciolek@mail.umcs.pl (U.I.M.); cbravo@uvigo.es (C.B.-D.)

Table S1. Synthetic routes of flavonoid-based cocrystals reported from 2017 to date in the CSD (accessed January 2025) and solvents employed. See text for a brief description of the methods. NG: neat grinding, LAG: liquid-assisted grinding, SE: solvent evaporation, SL: slurry, CC: cooling crystallization, AS: antisolvent process, GAS-gas antisolvent process, RC: reaction crystallization, OPD: one-pot deracemization. Abbreviations: (+) performed (a) indicates that an additional method was used to obtain single-crystal of cocrystal; (b) indicates that sonication used to dissolve; (c) indicates that various methods used to crystal preparation, and (d) indicates that solvent evaporation preceded by milling.

Flavonoid	Coformer	Solid-based methods									Solution based-methods																	
		NG	LAG								SE																	
			ethanol	acetonitrile	methanol	n-butanol	water/ethanol/acetone	Water/ethanol	isopropanol	acetone	methanol	water	acetonitrile	tetrahydrofuran	ethanol/THF	ethanol	propanol	ethanol/ethyl acetate	ethanol/dichloromethane	ethanol/acetone	dichloromethane/isopropanol	dichloromethane/ethyl acetate	acetonitrile/n-butanol/ethanol	water/methanol/ethyl acetate	acetone	methanol/ethyl acetate	ethyl acetae	ethanol/water
Flavone	Naringenin [1]	+[a]																										
	Diethylstilbestrol [2]										+			+														
	Sulfaguanidine [3][c]																											
	Dapsone [3][c]																											
	Sulfanilamide [3][c]																											
Chrysin	Cytosine [4]		+																									
	thiamine hydrochloride [4]		+																									
	berberine chloride [5]		+[a]																									
	4,4’-bipyridine [6]																											
	1,4-bis(pyridin-4-ylmethoxy)benzene [6]																											
	4,4′-vinylenedipyridine [7]														+[b]													
	Ethionamide [8]																						+[d]					
	isonicotinic acid [9][c]							+																				

Flavonoid	Coformer	Solution based-methods																												
		SL													CC				AS						GAS		RC			OPD
		ethanol/dichloromethane	methanol	acetonitrile	ethanol	ethyl acetate	isopropylalcohol	isopropyl ether	methanol/ethyl acetate	acetonitrile/ethyl acetate	acetone	dioxane	tetrahydrofuran	water	methanol	ethyl acetate	ethyl acetate/acetonitrile	dichloromethane/methyl isobutyl ketone/acetone	Ethanol/ethyl acetate + n-heptane	isopropanol+ n-heptane	dichloromethane/tetrahydrofuran + n-heptane	ethyl acetate + n-heptane	THF+hexane	DMSO+water	acetone	ethanol	methanol	ethanol	acetone/H_2O	
Flavone	Naringenin [1]																													
	Diethylstilbestrol [2]																													
	Sulfaguanidine [3][c]																													
	Dapsone [3][c]																													
	Sulfanilamide [3][c]																													
Chrysin	Cytosine [4]																													
	thiamine hydrochloride [4]																													
	berberine chloride [5]																													
	4,4’-bipyridine [6]															+[b]														
	1,4-bis(pyridin-4-ylmethoxy)benzene [6]																+[b]													
	4,4′-vinylenedipyridine [7]																													
	Ethionamide [8]																													
	isonicotinic acid [9][c]																													

Apigenin	4,4’-bipyridine [10,11] [c]		+[a]																										+[a]																												
	1,2-di(pyridin-4-yl)ethane [12]															+[a]																																									
	Carbamazepine [12]															+[a]																																									
	(DL)-proline [12]																	+[a]																																							
	δ-valerolactam [12]		+[a]																																																						
	D-proline [12]																	+[a]																																							
	L-proline [12]																	+[a]																																							
	Berberine [13]																													+[a]																											
	Piperazine [14]															+[a]																																									
	Theophylline [15]										+[a]																																															
	Nicotinamide [16]		+[a]																																																						
Luteolin	4,4′-vinylenedipyridine [7]																			+[b]																																					
	Isoniazid [17]		+																																																						
	Caffeine [17]		+																																																						
Fisetin	Caffeine [18]																																									+															
	Nicotinamide [18]																																									+															
kaempferol	Praziquantel [19]																														+[a]																										
	Olaparib [20]																				+																																				
	5-fluorouracil [21] [c]		+[a]																													+[a]																									
	4,4’-bipyridine [22,23]																			+																																					
	Propylthiouracil [24,25] [c]			+[a]							+																																														
	Aripiprazole [26]																																													+	+	+									
	Olanzapine [27]																																																+								
	Palbociclib [28]																																																								
Quercetin	Praziquantel [19]																																+[a]																								
	Theophylline [29] [c]				+																			+																																	
	Olaparib [20]																					+																																			
	Pentoxifylline [30]																																	+[a]																							

	Betaine [31]																																			+[a]																					
	Pyrazinamide [32]																							+																																	
	o-dianisidine [33]			+[a]																																																					
	1,10-phenanthroline [33]			+[a]																																																					
	4-(5-ethyl-1-benzofuran-2-yl)pyridine [33]			+[a]																																																					
	4-(1-benzofuran-2-yl)pyridine [33]			+[a]																																																					
	4,4′-vinylenedipyridine [7]															+[b]																																									
	4,4'-ethane-1,2-diyldipyridine [34]																													+[a]																											
	4,4′-azobispyridine [34]																													+[a]		+[a]						+[a]	+[a]	+[a]																	
	4,4’-bipyridine [23]																			+																																					
	Caffeine [35]																													+																											
	Isoniazid [36]				+																																																				
	Levofloxacin [37] [c]		+																												+																									
	Ciprofloxacin [37] [c]		+																												+																									
	Nicotinamide [38, 39]															+																																			+					
	L-proline [9, 10]															+																																				+				
	D-proline [9]															+																																									
	Benzamide [41] [c]	+	+																																																						
	Picolinamide [41][c]	+	+																																																						
	Isonicotinamide [41] [c]	+	+																																																						
	pyrazinoic acid [41] [c]	+	+																																																						
	succinic acid [42]										+																																														
	Arginine [43] [c]										+					+	+																																								
	isonicotinic acid [9] [c]							+								+																																									
	Piroxicam [9]							+																																																	
	4-dimethylaminopyridine [9]															+																																									
Baicalein	Ethionamide [44]		+[a]																																																						
	Metamitron [45]	+[a]																																																							

	Temozolomide [46]																													+																											
	Izoniazide [47]																																																					+			
	Isonicotinamide [47]																																																+				+[a]			
	Caffeine [35,47] [c]																													+																							+[a]			
	Theophylline [47] [c]																							+[b]																													+[a]		+[a]	
	4,4’-bipyridine [48,49]											+							+																																						
	4,4′-vinylenedipyridine [7]														+[b]																																										
	Pyrazinamide [50]					+[a]																																																			
	Betaine [31]				+[a]																					+																															
	Oxaliplatin [51]						+[a]																																																		
	Nicotinamide [52]																										+																														
Myricetin	Tegafur [53] [c]		+[a]																												+[a]																										
	Praziquantel [19]																																				+[a]																				
	4,4’-ethylenebispyridine [54]																															+[a]																									
	Caffeine [35,55]																													+[a]																											
	Berberine [56]																															+[a]																									
	4,4'-bipyridine [23,57]																			+																																						
	Isonicotinatmide [55]																													+[a]																											
	Proline [55]																															+																									
	Nicotinamide [55]																													+[a]																											
	L-carnitine [58]																																+[b]																								
	Levofloxacin [37] [c]		+																												+																									
dihydromyricetin	Berberine [56]																															+[a]																									
	Caffeine [59]																														+[a]																										
	Theophylline [59]																														+[a]																										
	4,4'-bipyridine [60]																			+																																						
	4,4'-(ethene-1,2-diyl)dipyridine [7]														+[b]																																										
	Pentoxifylline [61]																																								+																

	Diphenylprolinol [61]																																																							+
Dihydroquercetin	4,4'-bipyridine [60]																		+																																						
	Isonicotinamide [62]																									+																															
Naringenin	Metamitron [62]	+[a]																																																							
	4-(1H)pyridine [1]	+[a]																																																							
	Piperazine [1]	+[a]																																																							
	Anthranilamide [1]	+[a]																																																							
	4,4'-bipyridine [1]	+[a]																																																							
	Flavone [1]	+[a]																																																							
	permethylated β-cyclodextrin [63]											+																																													
	Isonicotinamide [64]																																+[a]																								
	Betaine [64]																															+[a]																									
	picolinic acid [64]																																+																								
	4,4'-(ethene-1,2-diyl)dipyridine (4,4′-vinylenedipyridine) [7]										+[b]																																														
	Carbamazepine [65,66] [c]		+													+																																		+						
	Oxaliplatin [51]						+[a]																																																		
	Lobaplatin [67]								+[a]																																																
	Norfloxacin [68]																		+																																						
	isonicotinic acid [9]															+[a]																																									
	Nicotinamide [9]							+																																																	
Hesperetin	4,4'-ethylenebispyridine [54]																															+[a]																									
	Tetraethylammonium [69]																													+[a]																											
	Sophocarpinium [70]																																		+																						
	Temozolomide [71] [c]																																																								
	Carbamazepine [72-74]																																																						+[a]		
	Pirfenidone [75]																																								+[a]																
	Caffeine [76]		+																																																						
	Nicotinamide [76]		+																																																						

	picolinic acid [76]		+[a]																																																						
	palmatine chloride [77] [c]																												+[a]		+[a]																									
	4,4′-vinylenedipyridine [7]														+[b]																																									
	Piperine [78]																																																				+[a]		
	Levofloxacin [37]		+																																																					
	4,4 ′-bipyridine [79] [c]																							+[a]						+[a]																										
	nicotinic acid [9]							+																																																
	4-dimethylaminopyridine [9]														+[a]																																									
Nobiletin	Urea [80]		+[a]																																																					
	oxalic acid [80]		+																																																					
	gallic acid [80]		+																																																					
	salicylic acid [80]		+																																																						
	formic acid [80]		+[a]																																																					
Daidzein	4,4’-bipyridine [81]																+[a]																																								
	Piperazine [82]																																																					+[a]		
	Berberine [83]				+[a]																																																				
	Isonicotinamide [84]		+																																																					
	Theobromine [84]		+																																																					
	Cytosine [84]		+																																																					
	Theophylline [85]										+																																													
Genistein	4,4’-ethylenebispyridine [54]																														+[a]																									
	4,4’-bipyridine [86]																		+																																						
	4,4′-vinylenedipyridine [7]														+[b]																																									
	Ligustrazine [87] [c]				+[a]																										+[a]																									
	Piperazine [88]									+[a]																																														
	Pirfenidone [75]																																								+[a]																
	Palmatine [89]																																																				+[a]			
	Theophylline [90]																																																								+

Pratensol	4,4’-ethylenebispyridine [54]																															+[a]																									
	4,4’-vinylenedipyridine [7]																			+[b]																																							
xanthohumol	Caffeine [91]												+																																												
	Nicotinamide [91]												+																																												
	glutarimide (piperidine-2,6-dione) [91]												+																																												
	Acetamide [91]												+																																												
Phloretin	Isoniazid [92]										+																																														
	Betaine [31]				+[a]																																																				
	β-cyclodextrin [93]																											+																													
	Nicotinamide [94,95] [c]				+[a]																									+[a]																											
	Isonicotinatmide [95]																													+[a]																											
Catechin	Barbituric acid [96]															+																																									
	Urea [97]	+[a]																																																							
L-epicatechin	4,4’-vinylenedipyridine [7]										+[b]																																														

Table S2. Techniques and methods commonly used to characterize flavonoid cocrystals and physicochemical and biological properties of the cocrystals reported in CSD from 2017 to date. SCXRD - single-crystal X-ray diffraction; PXRD – powder X-ray diffraction; IR - infrared spectroscopy; DSC - differential scanning calorimetry; PK - in vivo pharmacokinetic experiment; IDR - intrinsic dissolution rate; DVS - dynamic vapor sorption; SFS - solid state fluorescence spectra.

Flavonoid	Coformer	Diffraction methods			Spectroscopic methods								Microscopic methods						Thermal methods		Physicochemical properties																			Biological properties												Computational studies
		SCXRD	PXRD	VT-PXRD	IR	Raman	Solid state NMR	HNMR in solutiom	CNMR in solutiom	LC-MS	Solid UV-VIS	XPS	Polarizing Microscopy (PM)	Hot-Stage Microscopy (HSM)	Light Microscopy	optical microscope	SEM	TEM	TGA	DSC	Solubility	Saturation Solubility	Dissolution test	Selective dissolution experiments	Equilibrium solubility studies	Phase Solubility Analysis.	IDR	DVS	FluxMeasurements	Accelerated Stability studies	Hygroscopic test	Powder compaction	Specific surface area determination	Leaching experiments in the soil column	herbicidal activity	Tabletability	ternary phase diagram	Sublimation Kinetics.	SFS	PK	Antioxidant activity	Antihemolytic activity	Anti-inflammatory evaluation	Antibacterial properties	Hepatotoxicity Study	DPPH scavenging	ABTS scavenging	Cytotoxicity test	Antitumor activities	toxicity test	interaction of AP and AP-Nico pharmaceutical cocrystal with human serum albumin	
flavone	naringenin [1]	+	+		+															+	+																															
	diethylstilbestrol [2]	+	+										+						+	+							+													+												
	sulfaguanidine [2]		+											+					+	+																																
	dapsone [2]		+											+					+	+																																
	sulfanilamide[2]		+											+					+	+																																
chrysin	cytosine [4]		+		+		+													+					+		+													+	+	+	+									
	thiamine hydrochloride [4]		+		+		+													+					+		+													+	+	+	+									
	berberine chloride [5]	+	+																	+								+												+												+
	4,4’-bipyridine [6]	+	+		+														+	+			+																									+				
	1,4-bis(pyridin-4-ylmethoxy)benzene [6]	+	+		+														+	+			+																									+				
	4,4′-vinylenedipyridine [7]	+	+		+															+																																+
	ethionamide [8]	+	+		+														+	+			+				+													+												
apigenin	4,4’-bipyridine [10, 11]	+	+		+	+													+	+		+	+		+	+														+			+							+		
	1,2-di(pyridin-4-yl)ethane [12]	+	+		+										+				+	+			+		+																											
	carbamazepine [12]	+	+		+										+				+	+			+		+																											
	(DL)-proline [12]	+	+		+										+				+	+			+		+																											
	δ-valerolactam [12]	+	+		+										+				+	+			+		+																											
	D-proline [12]	+	+		+										+				+	+			+		+																											
	L-proline [12]	+	+		+										+				+	+			+		+																											
	berberine [13]	+																																																		
	piperazine [14]	+	+		+														+	+																																+
	theophylline [85]		+			+		+	+				+						+	+					+		+	+	+											+												
	nicotinamide [16]		+		+			+	+											+		+	+																												+	+
luteolin	4,4′-vinylenedipyridine [7]	+	+		+															+																																+
	isoniazid [17]		+		+			+												+		+																		+												+
	caffeine [17]		+		+			+												+		+																		+												+
fisetin	caffeine [18]		+		+												+			+		+					+													+			+									

	nicotinamide [18]		+		+												+			+		+					+															+			+										
kaempferol	praziquantel [19]	+	+		+															+						+																												+
	olaparib [20]	+	+		+												+		+	+			+				+			+																			+					+
	5-fluorouracil [21]		+		+												+		+	+			+					+		+																							
	4,4’-bipyridine [22, 23]	+	+		+			+	+										+	+											+													+									+
	propylthiouracil [24, 25]	+	+														+		+	+			+		+																												+
	aripiprazole [26]	+	+		+														+	+			+																													
	olanzapine [27]	+	+	+	+														+	+			+																													+
	palbociclib [28]	+			+												+		+	+			+							+		+																	+					+
quercetin	praziquantel [19]	+	+		+															+						+																												+
	theophylline [29]	+	+		+			+		+							+		+				+		+						+																					
	olaparib [20]	+	+		+												+		+	+			+				+			+																			+					+
	pentoxifylline [30]	+	+																+	+			+							+											+												
	betaine [31]	+	+				+													+			+					+		+	+																					
	pyrazinamide [32]	+																					+																	+												
	o-dianisidine [33]	+	+		+	+						+				+	+																																			
	1,10-phenanthroline [33]	+	+		+	+						+				+	+																																			
	4-(5-ethyl-1-benzofuran-2-yl)pyridine [33]	+	+		+	+						+				+	+																																			
	4-(1-benzofuran-2-yl)pyridine [33]	+	+		+	+						+				+	+																																			
	4,4′-vinylenedipyridine [7]	+	+		+															+																																	+
	4,4'-ethane-1,2-diyldipyridine [34]	+	+								+								+	+										+																							+
	4,4′-azobispyridine [34]	+	+								+								+	+										+																							+
	4,4’-bipyridine [23]	+	+		+			+	+																																			+									
	caffeine [35]		+		+															+	+																															+
	isoniazid [36]		+		+															+			+				+			+															+					+		
	levofloxacin [37]		+	+				+											+																									+									
	ciprofloxacin [37]		+	+				+											+																									+									
	nicotinamide [38, 39]		+		+	+											+		+	+			+																	+												
	L-proline [9, 40]		+			+											+			+			+										+																				+
	D-proline [9]		+																																																	+
	benzamide [41]		+		+														+																																	
	picolinamide [41]		+		+														+																																	
	isonicotinamide [41]		+		+														+																																	
	pyrazinoic acid [41]		+		+														+																																	
	succinic acid [42]		+		+												+			+			+																														
	arginine [43]		+		+	+							+				+			+		+																															
	isonicotinic acid [9]		+																																																	+
	4-dimethylaminopyridine [9]		+																																																	+
	piroxicam [9]		+																																																	+
baicalein	ethionamide [44]	+	+																	+		+					+													+					+					+		
	metamitron [45]	+	+			+													+	+			+		+					+				+	+																	+
	temozolomide [46]	+	+		+															+			+							+																						
	izoniazide [47]	+	+		+									+					+	+			+					+												+												
	isonicotinamide [47]	+	+		+									+					+	+			+					+												+												
	caffeine [35, 47]	+	+		+									+					+	+			+					+												+												+
	theophylline [47, 98]	+	+		+									+			+		+	+			+					+												+												
	4,4’-bipyridine [48, 49]	+	+		+														+				+																													
	4,4′-vinylenedipyridine [7]	+	+		+															+																																	+
	pyrazinamide [50]	+	+		+														+	+							+													+					+							
	betaine [31]	+	+				+													+			+					+		+	+																					
	oxaliplatin [51]	+	+		+														+	+		+	+																									+					+
	nicotinamide [52]		+														+			+			+																	+												
myricetin	tegafur [53]	+	+		+			+											+	+			+					+		+		+				+																
	praziquantel [19]	+	+		+															+						+																												+
	4,4’-ethylenebispyridine [54]	+	+		+														+	+			+																									+	+			
	caffeine [35, 55]	+	+		+															+			+				+																										+
	berberine [56]	+	+																+	+					+			+																					+				+
	4,4'-bipyridine [23, 57]	+	+					+	+																						+																						+
	isonicotinatmide [55]	+	+		+															+			+				+																										+
	Proline [55]	+	+		+															+			+				+																										+

	nicotinamide [55]	+	+		+																	+			+				+																										+
	L-carnitine [58]		+		+	+	+												+	+			+								+					+																
	levofloxacin [37]		+	+				+											+																									+								
dihydromyricetin	berberine [56]	+	+																+	+						+			+																				+				+
	caffeine [99]	+	+														+							+													+															+
	theophylline [99]	+	+														+							+													+															+
	4,4'-bipyridine [60]	+	+		+														+				+								+																					
	4,4'-(ethene-1,2-diyl)dipyridine [7]	+	+		+															+																																+
	pentoxifylline [61]	+	+																	+		+	+					+																				+				
	diphenylprolinol [100]	+																																																		
dihydroquercetin	4,4'-bipyridine [60]	+	+		+														+				+								+																					
naringenin	isonicotinamide [62]		+										+							+			+																	+												
	metamitron [45]	+	+			+													+	+			+		+					+				+	+																	+
	4-(1H)pyridone [1]	+	+		+															+		+																														
	piperazine [1]	+	+		+															+		+																														
	anthranilamide [1]	+	+		+															+		+																														
	4,4’-bipyridine [1]	+	+		+															+		+																														
	flavone [1]	+	+		+															+		+																														
	permethylated β-cyclodextrin [63]	+																																																		+
	isonicotinamide [64]	+	+		+		+	+												+			+																														
	betaine [64]	+	+		+		+	+												+			+																														
	picolinic acid [64]	+	+		+		+	+												+			+																														
	4,4'-(ethene-1,2-diyl)dipyridine [7]	+	+		+															+																																+
	carbamazepine[65, 66]	+	+		+		+	+									+		+	+		+			+		+				+								+	+												
	oxaliplatin [51]	+	+		+														+	+		+	+		+		+																					+					+
	lobaplatin [67]	+	+		+			+											+	+		+	+																										+			
	norfloxacin [68]	+	+		+			+									+	+		+											+													+						+			
	isonicotinic acid [9]	+	+																																																	+
	nicotinamide [9]		+																																																	+
hesperetin	4,4’-ethylenebispyridine [54]	+	+		+														+	+			+																									+	+			
	tetraethylammonium [69]	+	+	+															+	+																																
	sophocarpinium [70]	+	+		+														+	+					+		+	+		+																							+
	temozolomide [71]	+	+		+			+												+			+					+		+																						
	carbamazepine [72, 73, 101]		+		+	+									+		+		+	+			+																+														+
	pirfenidone [75]	+	+		+														+	+					+		+	+																								+
	caffeine [76]		+		+		+													+			+		+															+		+	+			+						
	nicotinamide [76]		+		+		+													+			+		+															+		+	+			+						
	picolinic acid [76]	+	+		+		+													+			+		+															+		+	+			+						
	palmatine chloride [76]	+	+		+														+	+		+	+						+																								+
	4,4′-vinylenedipyridine [7]	+	+		+															+																																+
	piperine [78]	+	+		+														+	+			+		+															+												
	levofloxacin [37]		+	+				+											+																									+								
	4,4 ′-bipyridine [79]	+	+		+															+					+																											+
	nicotinic acid [9]		+																																																	+
	4-dimethylaminopyridine [9]	+	+																																																	+
nobiletin	urea [80]	+	+																+	+			+																													
	oxalic acid [80]		+																+	+			+																													
	gallic acid [80]		+																+	+			+																													
	salicylic acid [80]]		+																+	+			+																													
	formic acid [80]	+	+																+	+			+																													
daidzein	4,4’-bipyridine [81]	+	+		+									+						+			+		+																											
	piperazine [82]	+	+		+														+	+			+		+				+	+										+													+
	berberine [83]	+																																																	
	isonicotinamide [83]		+		+		+													+					+		+			+										+		+	+			+						
	theobromine [83]		+		+		+													+					+		+			+										+		+	+			+						
	cytosine [83]		+		+		+													+					+		+			+										+		+	+			+						
	theophylline [85,90]		+			+		+	+				+						+	+					+		+	+	+	+											+													+
genistein	4,4’-ethylenebispyridine [54]	+	+		+														+	+			+																									+	+			
	4,4’-bipyridine [86]	+	+		+														+	+			+		+																				+								
	4,4′-vinylenedipyridine [7]	+	+		+															+																																	+

	ligustrazine [87]	+	+		+												+		+	+			+															+		+												+
	piperazine [88]	+	+		+														+	+			+							+										+												+
	pirfenidone [75]	+	+		+														+	+					+		+	+																								+
	palmatine [89]	+																																																		
pratensol	4,4’-ethylenebispyridine [54]	+	+		+														+	+			+																									+	+			
	4,4’-vinylenedipyridine [54]	+	+		+															+																																+
xanthohumol	caffeine [89, 91]	+	+		+	+										+							+		+																											+
	nicotynamide [89]	+	+		+	+										+							+		+																											+
	glutarimide (piperidine-2,6-dione) [91]	+	+		+	+										+							+		+																											+
	acetamide [91]	+	+		+	+										+							+		+																											+
phloretin	isoniazid [92]	+	+		+			+											+	+			+		+															+						+	+		+			+
	betaine [31]	+	+				+													+			+					+		+	+																					
	β-cyclodextrin [93]	+																																																		+
	nicotinamide [94, 95]	+	+			+							+						+	+			+		+		+	+											+														+
	isonicotinatmide [95]	+	+			+							+						+	+			+		+														+														+
catechin	barbituric acid [96]	+	+			+													+	+			+		+																											+
	urea [97]	+	+	+															+	+					+																											
L-epicatechin	4,4’-vinylenedipyridine [7]	+	+		+															+																																+

REFERENCES

[1] Khandavilli UBR, Skořepová E, Sinha AS, Bhogala BR, Maguire NM, Maguire AR, et al. Cocrystals and a Salt of the Bioactive Flavonoid: Naringenin. Crystal Growth & Design. 2018;18:4571-7.
[2] Li Z, Li M, Peng B, Zhu B, Wang J-r, Mei X. Improving Compliance and Decreasing Drug Accumulation of Diethylstilbestrol through Cocrystallization. Crystal Growth & Design. 2019;19:1942-53.
[3] Petrick TL, Grünwald A, Braun DE. Flavone Cocrystals: A Comprehensive Approach Integrating Experimental and Virtual Methods. Crystal Growth & Design. 2024;24:4195-212.
[4] Chadha R, Bhalla Y, Nandan A, Chadha K, Karan M. Chrysin cocrystals: Characterization and evaluation. J Pharm Biomed Anal. 2017;134:361-71.
[5] Sa R, Zhang Y, Deng Y, Huang Y, Zhang M, Lou B. Novel Salt Cocrystal of Chrysin with Berberine: Preparation, Characterization, and Oral Bioavailability. Crystal Growth & Design. 2018;18:4724-30.
[6] Pang X, Tao Y, Zhang J, Chen H, Sun A, Ren G, et al. New Chrysin-based co-crystals: Synthesis, characterization and dissolution studies. Journal of Molecular Structure. 2023;1271:134079.
[7] Zhang Y, Zhu B, Ji W-J, Guo C-Y, Hong M, Qi M-H, et al. Insight into the Formation of Cocrystals of Flavonoids and 4,4′-Vinylenedipyridine: Heteromolecular Hydrogen Bonds, Molar Ratio, and Structural Analysis. Crystal Growth & Design. 2021;21:2720-33.
[8] Li J-Y, Liu F, Li Y-Y, Bao X-Y, Li Y, Li Y-T, et al. Hepatoprotective cocrystal of ethionamide: A new attempt to refurbish old drug through crystal engineering. Journal of Crystal Growth. 2022;582:126523.
[9] Li D, Ning Z, Gong Z, Zhou L, Xu L, He F, et al. Insights into salts/cocrystals formation of polyhydroxy natural products as well as their separation behavior via cocrystallization. Separation and Purification Technology. 2025;361:131453.
[10] Ma X-Q, Zhuang C, Wang B-C, Huang Y-F, Chen Q, Lin N. Cocrystal of Apigenin with Higher Solubility, Enhanced Oral Bioavailability, and Anti-inflammatory Effect. Crystal Growth & Design. 2019;19:5531-7.
[11] Yue H, Wang J. Machine Learning Prediction of Flavonoid Cocrystal Formation Combined with Experimental Validation. Industrial & Engineering Chemistry Research. 2023;62:20767-76.
[12] Makadia J, Seaton CC, Li M. Apigenin Cocrystals: From Computational Prescreening to Physicochemical Property Characterization. Crystal Growth & Design. 2023;23:3480-95.
[13] Yanjie Z, Benyong L, Yali H, Xiaodong H, Huazhong W. Crystal structure of 9,10-dimethoxy-5,6-dihydro-[1,3]dioxolo[4,5-g]isoquinolino[3,2-a]isoquinolin-7-ium 5-hydroxy-2-(4-hydroxyphenyl)-4-oxo-4H-chromen-7-olate trihydrate, C35H33NO12. Zeitschrift für Kristallographie - New Crystal Structures. 2018;233:865-7.
[14] Zhang J, Shen R, Zhang X, Li G, Wang X. A pharmaceutical cocrystal of apigenin with piperazine: Preparation, structural characterization, and dissolution performance. Journal of Molecular Structure. 2024;1298:137027.
[15] Huang S, Xue Q, Xu J, Ruan S, Cai T. Simultaneously Improving the Physicochemical Properties, Dissolution Performance, and Bioavailability of Apigenin and Daidzein by Co-Crystallization With Theophylline. J Pharm Sci. 2019;108:2982-93.
[16] Sun Y, Guo M. Imaging supermolecular interactions of the pharmaceutical-cocrystal of apigenin-nicotinamide binding with serum albumin. 2023;47:554-71.
[17] Luo Y, Chen S, Zhou J, Chen J, Tian L, Gao W, et al. Luteolin cocrystals: Characterization, evaluation of solubility, oral bioavailability and theoretical calculation. Journal of Drug Delivery Science and Technology. 2019;50:248-54.

[18] Mohite R, Mehta P, Arulmozhi S, Kamble R, Pawar A, Bothiraja C. Synthesis of fisetin co-crystals with caffeine and nicotinamide using the cooling crystallization technique: biopharmaceutical studies. New Journal of Chemistry. 2019;43:13471-9.
[19] Yang D, Cao J, Heng T, Xing C, Yang S, Zhang L, et al. Theoretical Calculation and Structural Analysis of the Cocrystals of Three Flavonols with Praziquantel. Crystal Growth & Design. 2021;21:2292-300.
[20] Duan C, Chen Y, Zhang Y, Liang F, Liu W, Xiao X, et al. Two Cocrystals of Olaparib with Flavonoids toward Sustained Release: Structure, Dissolution Behavior, and Anticancer Activity Analysis. Crystal Growth & Design. 2022;22:4885-94.
[21] Lv W-T, Liu X-X, Dai X-L, Long X-T, Chen J-M. A 5-fluorouracil–kaempferol drug–drug cocrystal: a ternary phase diagram, characterization and property evaluation. CrystEngComm. 2020;22:8127-35.
[22] Su X, Zhang Y-n, Yin H-m, Liu L-x, Zhang Y, Wu L-l, et al. Preparation of a 1:1.5 cocrystal of kaempferol with 4,4′-bipyridine based on analyzing intermolecular interaction of building units. Journal of Molecular Structure. 2019;1177:107-16.
[23] Zhang Y-N, Yin H-M, Zhang Y, Zhang D-J, Su X, Kuang H-X. Cocrystals of kaempferol, quercetin and myricetin with 4,4′-bipyridine: Crystal structures, analyses of intermolecular interactions and antibacterial properties. Journal of Molecular Structure. 2017;1130:199-207.
[24] Xiao Y, Zhou L, Hao H, Bao Y, Yin Q, Xie C. Cocrystals of Propylthiouracil and Nutraceuticals toward Sustained-Release: Design, Structure Analysis, and Solid-State Characterization. Crystal Growth & Design. 2021;21:1202-17.
[25] Xiao Y, Yang W, Zhou L, Hao H, Bao Y, Yin Q, et al. Growth mechanism of the spherulitic propylthiouracil–kaempferol cocrystal: new perspectives into surface nucleation. CrystEngComm. 2021;23:2367-75.
[26] Liu W, Ma R, Liang F, Duan C, Zhang G, Chen Y. New Cocrystals of Antipsychotic Drug Aripiprazole: Decreasing the Dissolution through Cocrystallization. Molecules. 2021;26.
[27] Liang F, Tan X, Hao S, Liu W, Duan C, Zhang G, et al. Synthesis and structural characterization of two novel olanzapine cocrystals with decreased or enhanced dissolution rate. Journal of Molecular Structure. 2022;1255:132340.
[28] Zhou H, Duan C, Qin H, Huang C, Hou J, Chen Y, et al. Synthesis and structural characterization of a novel palbociclib-kaempferol cocrystal with improved tabletability and synergistic antitumor activity. Journal of Molecular Structure. 2023;1281:135101.
[29] Wang L, Li S, Xu X, Xu X, Wang Q, Li D, et al. Drug-drug cocrystals of theophylline with quercetin. Journal of Drug Delivery Science and Technology. 2022;70:103228.
[30] Haskins MM, Kavanagh ON. Tuning the Pharmacokinetic Performance of Quercetin by Cocrystallization. Crystal Growth & Design. 2023;23:6059-66.
[31] Zhang Z, Li D, Luo C, Huang C, Qiu R, Deng Z, et al. Cocrystals of Natural Products: Improving the Dissolution Performance of Flavonoids Using Betaine. Crystal Growth & Design. 2019;19:3851-9.
[32] Liu F, Wang L-Y, Li Y-T, Wu Z-Y, Yan C-W. Protective Effects of Quercetin against Pyrazinamide Induced Hepatotoxicity via a Cocrystallization Strategy of Complementary Advantages. Crystal Growth & Design. 2018;18:3729-33.
[33] Maciołek U, Mendyk E, Kuśmierz M, Kozioł AE. Binary Co-Crystals of Quercetin: Synthesis, Structure, and Spectroscopic Characterization. ChemPlusChem. 2023;88:e202300166.
[34] Li Z, Zhou J, Zhang K, Zhang Y, Wu S, Gong J. Playing with Isostructurality from Binary Cocrystals to Ternary Cocrystal Solvates of Quercetin: Tuning Colors of Pigment. Crystal Growth & Design. 2022;22:5322-34.

[35] Xia Y, Wei Y, Chen H, Qian S, Zhang J, Gao Y. Competitive cocrystallization and its application in the separation of flavonoids. IUCrJ. 2021;8:195-207.
[36] Yadav B, Gunnam A, Thipparaboina R, Nangia AK, Shastri NR. Hepatoprotective Cocrystals of Isoniazid: Synthesis, Solid State Characterization, and Hepatotoxicity Studies. Crystal Growth & Design. 2019;19:5161-72.
[37] Fiore C, Antoniciello F, Roncarati D, Scarlato V, Grepioni F, Braga D. Levofloxacin and Ciprofloxacin Co-Crystals with Flavonoids: Solid-State Investigation for a Multitarget Strategy against Helicobacter pylori. Encapsulation of Natural Polyphenolic Compounds; a Review. 2024;16:203.
[38] Wu N, Zhang Y, Ren J, Zeng A, Liu J. Preparation of quercetin–nicotinamide cocrystals and their evaluation under in vivo and in vitro conditions. RSC Advances. 2020;10:21852-9.
[39] Dias JL, Rebelatto EA, Hotza D, Bortoluzzi AJ, Lanza M, Ferreira SRS. Production of quercetin-nicotinamide cocrystals by gas antisolvent (GAS) process. The Journal of Supercritical Fluids. 2022;188:105670.
[40] Dias JL, Rebelatto EA, Lanza M, Ferreira SRS. Production of quercetin-proline cocrystals by means of supercritical CO2 antisolvent. Advanced Powder Technology. 2023;34:104222.
[41] Souza FZRd, Almeida ACd, Ferreira PO, Fernandes RP, Caires FJ. Screening of coformers for quercetin cocrystals through mechanochemical methods. Eclética Química. 2022;47:64-75.
[42] Setyawan D, Oktavia IP, Farizka R, Sari R. Physicochemical Characterization and In Vitro Dissolution Test of Quercetin-Succinic Acid Co-crystals Prepared Using Solvent Evaporation. Turkish journal of pharmaceutical sciences. 2017;14:280-4.
[43] Mishra M, Agrawal S. Effect of stoichiometry upon the characteristics of quercetin-arginine cocrystals formulated through solution crystallization. Drug development and industrial pharmacy. 2024;50:163-72.
[44] Wang W-X, Liu F, Li J-Y, Xue J, Li Y-T, Liu R-M. A cocrystal for effectively reducing the hepatotoxicity of ethionamide. Journal of Molecular Structure. 2021;1243:130729.
[45] Xiao Y, Wu C, Zhou L, Yin Q, Yang J. Cocrystal engineering strategy for sustained release and leaching reduction of herbicides: a case study of metamitron. Green Chemistry. 2022;24:8088-99.
[46] Li J-M, Dai X-L, Li G-J, Lu T-B, Chen J-M. Constructing Anti-Glioma Drug Combination with Optimized Properties through Cocrystallization. Crystal Growth & Design. 2018;18:4270-4.
[47] Zhu B, Zhang Q, Wang J-R, Mei X. Cocrystals of Baicalein with Higher Solubility and Enhanced Bioavailability. Crystal Growth & Design. 2017;17:1893-901.
[48] Cheng G-L, Jiang C-J, Xia Y-F. The crystal structure of 4,4′-bipyridine-5,6,7-trihydroxy-2-phenyl-4H-chromen-4-one-water(1/2/2), C40H32N2O12. Zeitschrift für Kristallographie - New Crystal Structures. 2022;237:933-5.
[49] Liu L-x, Su X, Zhang Y-n, Yin H-m, Zhang Q, Feng Y-r, et al. A Cocrystal of Baicalein and 4,4′-Bipyridine with Zipper-Type Architecture. Journal of Chemical Crystallography. 2021;51:363-71.
[50] Hao S-Y, Li J-Y, Mu C-Q, Liu D-S, Yang Y, Li Y-T, et al. Hepatoprotective Pyrazinamide–Baicalein Cocrystal with a Rare Ratio of 7:3. Crystal Growth & Design. 2023;23:885-91.
[51] Yin H-M, Wu N, Zhou B-J, Hong M-H, Zhu B, Qi M-H, et al. Slow-Release Drug–Drug Cocrystals of Oxaliplatin with Flavonoids: Delaying Hydrolysis and Reducing Toxicity. Crystal Growth & Design. 2021;21:75-85.

[52] Pi J, Wang S, Li W, Kebebe D, Zhang Y, Zhang B, et al. A nano-cocrystal strategy to improve the dissolution rate and oral bioavailability of baicalein. Asian J Pharm Sci. 2019;14:154-64.
[53] Zhang M, Gu D-L, Zhen J-F, Lu T-B, Dai X-L, Chen J-M. A novel drug–drug cocrystal of tegafur and myricetin: optimized properties of dissolution and tabletability. CrystEngComm. 2023;25:6171-9.
[54] Zhang Y, Yang R, Yin H-M, Zhou B, Hong M, Zhu B, et al. Cocrystals of flavonoids with 4,4′-ethylenebispyridine: Crystal structures analysis, dissolution behavior, and anti-tumor activity. Journal of Molecular Structure. 2022;1252:132150.
[55] Ren S, Liu M, Hong C, Li G, Sun J, Wang J, et al. The effects of pH, surfactant, ion concentration, coformer, and molecular arrangement on the solubility behavior of myricetin cocrystals. Acta pharmaceutica Sinica B. 2019;9:59-73.
[56] Li P, Ramaiah T, Zhang M, Zhang Y, Huang Y, Lou B. Two Cocrystals of Berberine Chloride with Myricetin and Dihydromyricetin: Crystal Structures, Characterization, and Antitumor Activities. Crystal Growth & Design. 2020;20:157-66.
[57] Liu L, Liu M, Zhang Y, Feng Y, Wu L, Zhang L, et al. The role of hydroxyl group of ethanol in the self-assembly of pharmaceutical cocrystal of myricetin with 4,4′-bipyridine. Journal of Molecular Structure. 2022;1250:131848.
[58] Pang Z, Weng X, Wei Y, Gao Y, Zhang J, Qian S. Modification of hygroscopicity and tabletability of l-carnitine by a cocrystallization technique. CrystEngComm. 2021;23:2138-49.
[59] Sun J, Wang Y, Tang W. Enantioselectivity of chiral dihydromyricetin in multicomponent solid solutions regulated by subtle structural mutation. 2023;10:164-76.
[60] Liu L, Liu M, Zhang Y, Yin H, Su X, Zhang Q, et al. The role of 3-OH in the self-assembly of pharmaceutical cocrystals of dihydroflavonol with 4,4′-bipyridine. New Journal of Chemistry. 2021;45:1626-33.
[61] Liu L, Li Y, Zhang M, Zhang Y, Lou B. A Drug-Drug Cocrystal of Dihydromyricetin and Pentoxifylline. J Pharm Sci. 2022;111:82-7.
[62] Jiang J, Wang A, Zhang X, Wang Y, Wang Q, Zhai M, et al. The isonicotinamide cocrystal promotes inhibitory effects of naringenin on nonalcoholic fatty liver disease in mice. Journal of Drug Delivery Science and Technology. 2020;59:101874.
[63] Papaioannou A, Christoforides E, Bethanis K. Inclusion Complexes of Naringenin in Dimethylated and Permethylated β-Cyclodextrins: Crystal Structures and Molecular Dynamics Studies. Crystals. 2020;10:10.
[64] Luo C, Liang W, Chen X, Wang J, Deng Z, Zhang H. Pharmaceutical cocrystals of naringenin with improved dissolution performance. CrystEngComm. 2018;20:3025-33.
[65] Lee C, Cho AY, Yoon W, Yun H, Kang JW, Lee J. Cocrystal Formation via Resorcinol–Urea Interactions: Naringenin and Carbamazepine. Crystal Growth & Design. 2019;19:3807-14.
[66] Zhou F, Zhou J, Zhang H, Tong HHY, Nie J, Li L, et al. Structure determination and in vitro/vivo study on carbamazepine-naringenin (1:1) cocrystal. Journal of Drug Delivery Science and Technology. 2019;54:101244.
[67] Yin H-M, Xie J-Y, Jiang J-Y, Hong M, Zhu B, Ren G-B, et al. Strategy to Tune the Performance of Two Drug Components: Drug–Drug Cocrystals of Lobaplatin with Flavonoids. Crystal Growth & Design. 2022;22:2602-10.
[68] Zeng X, Zheng C, Qiu S, Jiang X, Wang X, Qian K, et al. Cocrystal of Naringenin and Norfloxacin: Crystal Transformation, Solubility, and Antibacterial and Anticancer Activities. Crystal Growth & Design. 2024;24:4416-27.
[69] Jin S, Haskins MM, Andaloussi YH, Ouyang R, Gong J, Zaworotko MJ. Conformational Trimorphism in an Ionic Cocrystal of Hesperetin. Crystal Growth & Design. 2022;22:6390-7.

[70] Wang Y, Qian Y-H, Hong M-H, Zhu B, Ren G-B, Qi M-H. Preparation, characterization, and crystal structures of novel sophocarpine salts with improvements on stability and solubility. Journal of Molecular Structure. 2023;1279:134992.
[71] Wang J, Dai X-L, Lu T-B, Chen J-M. Temozolomide–Hesperetin Drug–Drug Cocrystal with Optimized Performance in Stability, Dissolution, and Tabletability. Crystal Growth & Design. 2021;21:838-46.
[72] Liu L, Wang S, Ouyang J, Chen M, Zhou L, Liu Z, et al. Sustainable preparation of spherical particles of novel carbamazepine-hesperetin cocrystal via different crystallization strategies: From mechanism to application. Separation and Purification Technology. 2023;327:124954.
[73] Ouyang J, Liu L, Li Y, Chen M, Zhou L, Liu Z, et al. Cocrystals of carbamazepine: Structure, mechanical properties, fluorescence properties, solubility, and dissolution rate. Particuology. 2024;90:20-30.
[74] Ouyang J, Liu L, Ning Z, Gong Z, Zhou L, He F, et al. Fabrication of micro spherulitic particles of carbamazepine-hesperetin cocrystal via QESD with enhanced manufacturability and dissolution. Particuology. 2024.
[75] Meng L, Li D, Zhu Y, Wang J, Deng Z, Zhang H. Pirfenidone–flavonoid cocrystals with reduced solubility and dissolution rate. CrystEngComm. 2023;25:5133-40.
[76] Chadha K, Karan M, Bhalla Y, Chadha R, Khullar S, Mandal S, et al. Cocrystals of Hesperetin: Structural, Pharmacokinetic, and Pharmacodynamic Evaluation. Crystal Growth & Design. 2017;17:2386-405.
[77] Zhang Y, Li Y, Liu L, Guo Q, Sa R, Zhang M, et al. Two Cocrystal Polymorphs of Palmatine Chloride with Racemic Hesperetin. Crystal Growth & Design. 2022;22:1073-82.
[78] Liu Y, Yang F, Zhao X. Crystal Structure, Solubility, and Pharmacokinetic Study on a Hesperetin Cocrystal with Piperine as Coformer. Encapsulation of Natural Polyphenolic Compounds; a Review. 2022;14.
[79] Zhu Y, Wang L, Li J, Shi X, Deng Z, Zhang H. Hesperetin–4,4′-bipyridine cocrystal: Polymorphism, crystal structures, and thermodynamic relationship. Journal of Molecular Structure. 2025;1320:139719.
[80] Tokunaga S, Uchikoshi C, Hayashi K, Suzuki H, Ito M, Noguchi S. Novel Pharmaceutical Cocrystals and Solvate Crystals of Nobiletin, a Citrus Flavonoid with Potent Pharmacological Activity. Chemical & pharmaceutical bulletin. 2023;71:633-40.
[81] Bolus L, Wang K, Pask C, Lai X, Li M. Cocrystallisation of Daidzein with pyridine-derived molecules: Screening, structure determination and characterisation. Journal of Molecular Structure. 2020;1222:128893.
[82] Wang Z, Li S, Li Q, Wang W, Liu M, Yang S, et al. A Novel Cocrystal of Daidzein with Piperazine to Optimize the Solubility, Permeability and Bioavailability of Daidzein. Molecules. 2024;29:1710.
[83] Lou B, Huang Y, Zheng G, Lin Q. Crystal structure of 5,6-Dihydro-9,10-dimethoxybenzo[g]-1,3-benzodioxolo[5,6-a]chinolizinium 3-(4-hydroxyphenyl)-4-oxo-4H-chromen-7-olate - methanol - water (1/1/1), C36H33NO10. Zeitschrift für Kristallographie - New Crystal Structures. 2017;232:913-5.
[84] Bhalla Y, Chadha K, Chadha R, Karan M. Daidzein cocrystals: An opportunity to improve its biopharmaceutical parameters. Heliyon. 2019;5:e02669.
[85] Huang S, Xue Q, Xu J, Ruan S, Cai T. Simultaneously Improving the Physicochemical Properties, Dissolution Performance, and Bioavailability of Apigenin and Daidzein by Co-Crystallization With Theophylline. J Pharm Sci. 2019;108:2982-93.
[86] Zhang Y-N, Yin H-M, Zhang Y, Zhang D-J, Su X, Kuang H-X. Preparation of a 1:1 cocrystal of genistein with 4,4′-bipyridine. Journal of Crystal Growth. 2017;458:103-9.

[87] Li X, Liu X, Song J, Wang C, Li J, Liu L, et al. Drug–Drug Cocrystallization Simultaneously Improves Pharmaceutical Properties of Genistein and Ligustrazine. Crystal Growth & Design. 2021;21:3461-8.
[88] Wang Z, Li Q, An Q, Gong L, Yang S, Zhang B, et al. Optimized solubility and bioavailability of genistein based on cocrystal engineering. Natural Products and Bioprospecting. 2023;13:30.
[89] Zhang Y, Lou B, Huang Y, Zheng G, Lin Q. Crystal structure of 2,3,9,10-tetramethoxy-5,6-dihydroisoquinolino[2,1-b]isoquinolin-7-ium 5-hydroxy-3-(4-hydroxyphenyl)-4-oxo-4H-chromen-7-olate methanol solvate, C37H35N1O10. Zeitschrift für Kristallographie - New Crystal Structures. 2017;232:681-3.
[90] Zhu J, Yao H, Lu Y, Lu H, Liu Z, Wang L, et al. Theoretical exploration on the molecular configurations, solubilities and chemical reactivities of four flavonoid-based co-crystals. Journal of Molecular Liquids. 2023;376:121484.
[91] Budziak I, Arczewska M, Kamiński DM. Formation of Prenylated Chalcone Xanthohumol Cocrystals: Single Crystal X-ray Diffraction, Vibrational Spectroscopic Study Coupled with Multivariate Analysis. Molecules. 2019;24:4245.
[92] Lu Z, Chen H, Mo J, Yuan X, Wang D, Zheng X, et al. Cocrystal of phloretin with isoniazid: preparation, characterization, and evaluation. RSC Advances. 2023;13:10914-22.
[93] Aree T. How cyclodextrin encapsulation improves molecular stability of apple polyphenols phloretin, phlorizin, and ferulic acid: Atomistic insights through structural chemistry. Food Chem. 2023;409:135326.
[94] Aitipamula S, Shan LP, Gupta KM. Polymorphism and distinct physicochemical properties of the phloretin–nicotinamide cocrystal. CrystEngComm. 2022;24:560-70.
[95] Huang S, Xu J, Peng Y, Guo M, Cai T. Facile Tuning of the Photoluminescence and Dissolution Properties of Phloretin through Cocrystallization. Crystal Growth & Design. 2019;19:6837-44.
[96] Budziak-Wieczorek I, Maciołek U. Synthesis and Characterization of a (−)-Epicatechin and Barbituric Acid Cocrystal: Single-Crystal X-ray Diffraction and Vibrational Spectroscopic Studies. ACS Omega. 2021;6:8199-209.
[97] Leng F, Robeyns K. Urea as a Cocrystal Former-Study of 3 Urea Based Pharmaceutical Cocrystals. 2021;13.
[98] Li W, Pi J, Zhang Y, Ma X, Zhang B, Wang S, et al. A strategy to improve the oral availability of baicalein: The baicalein-theophylline cocrystal. Fitoterapia. 2018;129:85-93.
[99] Sun J, Wang Y, Tang W. Enantioselectivity of chiral dihydromyricetin in multicomponent solid solutions regulated by subtle structural mutation. IUCrJ. 2023;10:164-76.
[100] Su X, Sun J, Liu J, Wang Y, Wang J, Tang W, et al. Bifunctional Chiral Agent Enables One-pot Spontaneous Deracemization of Racemic Compounds. Angewandte Chemie International Edition. 2024;63:e202402886.
[101] Ouyang J, Liu L, Ning Z, Gong Z, Zhou L, He F, et al. Fabrication of micro spherulitic particles of carbamazepine-hesperetin cocrystal via QESD with enhanced manufacturability and dissolution. Particuology. 2024;93:11-21.
